# Aligned P(VDF-TrFE) Nanofibers for Enhanced Piezoelectric Directional Strain Sensing

**DOI:** 10.3390/polym10040364

**Published:** 2018-03-25

**Authors:** Yonggang Jiang, Longlong Gong, Xiaohe Hu, Yong Zhao, Huawei Chen, Lin Feng, Deyuan Zhang

**Affiliations:** 1 School of Mechanical Engineering and Automation, Beihang University, Beijing 100191, China; jiangyg@buaa.edu.cn (Y.J.); gong.ll@buaa.edu.cn (L.G.); huxh0706@buaa.edu.cn (X.H.); Chenhw75@buaa.edu.cn (H.C.); zhangdy@buaa.edu.cn (D.Z.); 2 Laboratory of Bio-inspired Smart Interfacial Science and Technology of the Ministry of Education, School of Chemistry, Beihang University, Beijing 100191, China; zhaoyong@buaa.edu.cn; 3 International research institute of Multidisciplinary Science, Beihang University, Beijing 100191, China

**Keywords:** piezoelectric polymer, P(VDF-TrFE), nanofibers, directional sensing

## Abstract

Piezoelectric poly(vinylidene fluoride-trifluoroethylene) (P(VDF-TrFE)) nanofibers fabricated by electrospinning have drawn increasing levels of attention in the fields of flexible sensors and nanogenerators. However, the directional dependence of piezoelectricity of electrospun nanofibers remains elusive. In this study, the piezoelectric performances of individual nanofibers are characterized by piezoresponse force microscopy (PFM), while the effects of annealing on β-phase crystallinities are investigated by X-ray diffraction (XRD) and Fourier transform infrared (FTIR) spectroscopy. The experimental results reveal that the as-spun P(VDF-TrFE) nanofibers form higher content of β-phase compared with spin-coated films, and the content of β-phase increases by annealing. The annealed P(VDF-TrFE) nanofiber exhibits distinct vertical polarization switching characteristics. The high piezoelectric output in the thickness direction and low piezoelectric output in the longitudinal direction of the nanofiber mats further confirm that the preferential dipole orientation of electrospun P(VDF-TrFE) nanofibers is normal to the surface of the substrate. Highly aligned P(VDF-TrFE) nanofibers show directional strain sensing ability due to the piezoelectric and mechanical anisotropy.

## 1. Introduction

Piezoelectric polymers and associated nanostructured materials are widely used in mechanical sensing and energy harvesting for their abilities to accumulate electrical charges in response to applied mechanical stresses [1,2,3,4]. Polymeric piezoelectric materials such as polyvinylidene fluoride (PVDF) and poly(vinylidene fluoride-trifluoroethylene) (P(VDF-TrFE)) have attracted significant attention for their flexibilities and biocompatibilities in contrast to the inherent brittleness of inorganic piezoelectric materials, such as barium titanate (BaTiO_3_), zinc oxide (ZnO), and lead zirconate titanate (PZT). Initially, PVDF and P(VDF-TrFE) devices that were processed into films as the sensing materials in micro-electro-mechanical systems (MEMS) [5,6,7,8,9,10]. In recent years, due to their enhanced piezoelectric performance, PVDF and P(VDF-TrFE) nanofibers fabricated by electrospinning process have shown tremendous potential applications for microsensors [11,12,13,14,15] and nanogenerators [16,17,18,19,20,21,22]. For example, a high-sensitivity acoustic sensor for the detection of low-frequency sound was developed using a PVDF nanofiber web that showed a remarkable mechanical to electrical energy conversion ability [12]. A wave-shaped piezoelectric/triboelectric hybrid nanogenerator was fabricated by sandwiching piezoelectric P(VDF-TrFE) nanofibers between wave-shaped Kapton films, resulting in higher outputs than individual piezoelectric or triboelectric nanogenerators [17]. Though various P(VDF-TrFE) nanofibers based devices have been reported, the significance of fiber alignment for strain sensing was not well evaluated. 

The piezoelectric performance of a P(VDF-TrFE) nanofiber is determined by its fabrication process and morphology. Compared with randomly oriented nanofibers, well aligned arrangements of such nanofibers can improve piezoelectric performance. Lin et al. proposed a near-field electrospinning (NFES) method for the fabrication of aligned PVDF and P(VDF-TrFE) nanofibers [23,24]. The NFES process generates electrical dipoles aligned along the axial direction of the nanofibers [25]. Interdigital electrodes have been used to increase the piezoelectric output of aligned nanofibers due to their poor abilities to produce high volumetric densities. As a result, a re-poling process is required to obtain piezoelectric polarizations in alternating dipole directions [26,27,28]. Far-field electrospinning using a rotating drum as the collector is an alternative approach for the fabrication of aligned nanofibers. The electrospinning process produces highly aligned nanofibers in large quantities with enhanced concentrations of the β-phase, as mechanical stretching result in α-phase transition to β-phase [29]. Only few studies have shown the thermo-electromechanical and piezoelectric performance of P(VDF-TrFE) nanofiber-based devices [30,31,32]. The highest piezoelectric charge constant of P(VDF-TrFE) nanofibers reported in previous studies was 35.5 pm/V, which were fabricated by the electrospinning process and followed by annealing at 135 °C [33]. The annealing process is a crucial processing factor to improve the crystallinity and the performances of polymers for sensing applications [34,35,36,37,38]. The piezoelectricity of P(VDF-TrFE) is determined not only by the crystallinity and fraction of the β-phase, but also the orientations of the dipoles in the β-phase. Mandal et al. demonstrated that the CF_2_ dipoles in the P(VDF-TrFE) nanofibers web prefer to be oriented perpendicular to the nanoweb surface; this preference has been verified by polarized Fourier transform infrared spectroscopy (FTIR) as well as piezoelectric signal detection in a nano-pressure sensor [31]. However, for aligned P(VDF-TrFE) nanofibers, the preferential dipole orientation and the directional piezoelectric performance need to be clarified for practical device applications. In this study, we report a detailed investigation on the piezoelectric responses of aligned P(VDF-TrFE) nanofibers. Highly aligned P(VDF-TrFE) nanofibers exhibit enhanced directional strain sensing performance. These findings are significant for the design and fabrication of flexible piezoelectric sensing devices using as-spun P(VDF-TrFE) nanofibers.

## 2. Materials and Methods

### 2.1. Materials

P(VDF-TrFE)-copolymer powder with a 75/25 mol % VDF/TrFE ratio, was purchased from Kureha Chemical Industries (Osaka, Japan). Dimethylformamide (DMF) which was used as solvent for the P(VDF-TrFE), was obtained from Tianjin Guangfu Science and Technology Co., Ltd (Tianjin, China). Acetone was supplied by Beijing Chemical Works (Beijing, China) and was used to dilute the mixture of P(VDF-TrFE) and DMF.

### 2.2. Fabrication of the P(VDF-TrFE) Nanofibers

**Figure 1:**
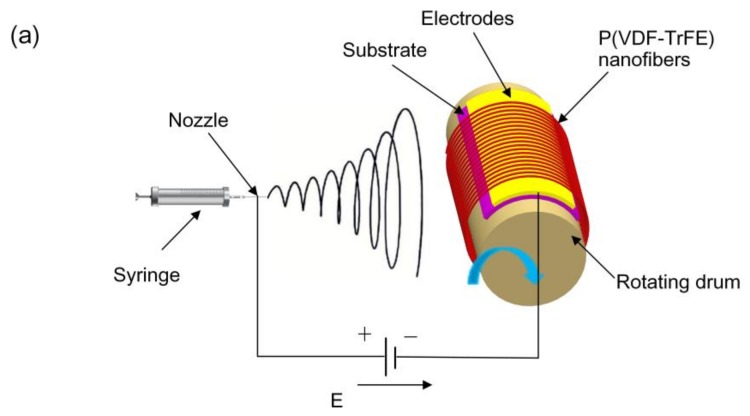

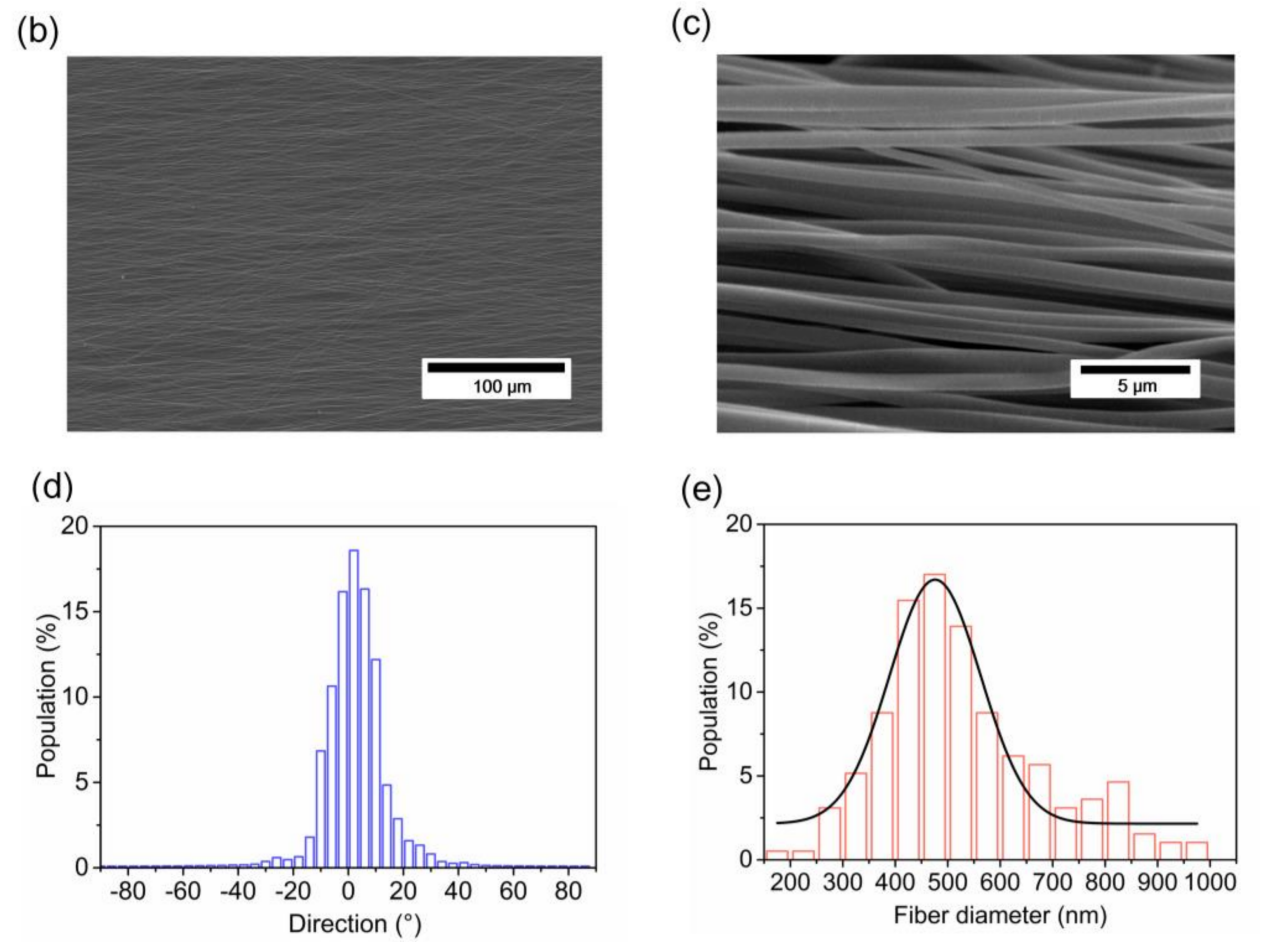
(**a**) Schematic illustration of the far-field electrospinning setup with a rotating-drum collector for the fabrication of highly aligned poly(vinylidene fluoride-trifluoroethylene) (P(VDF-TrFE)) nanofibers. (**b**) Scanning electron microscopy (SEM) micrograph of aligned P(VDF-TrFE) nanofibers. (**c**) Enlarged SEM micrograph of aligned P(VDF-TrFE) nanofibers. (**d**) The degree of nanofiber alignment. (**e**) Typical fiber diameter distribution and fit by a Gaussian curve.

The P(VDF-TrFE) powder was dissolved in dimethylformamide/acetone (3:2 *w/w*; DMF/acetone) by mechanical stirring for 12 hours at room temperature. The weight fraction of P(VDF-TrFE) in the solvent was 21 wt %. A typical far-field electrospinning setup is shown in Figure 1a. A 1.0 mL plastic syringe fitted with a 27 G stainless steel nozzle was fed by a syringe pump at a flow rate of 1–1.5 mL/h. The positive lead from a high-voltage supply was connected to the metal nozzle, and a high voltage of around 12 kV with a separation of about 7.5 cm between the nozzle tip and grounded electrodes was applied. The electrodes were fixed on the rotating drum with a diameter of 10 cm, which was rotated at a rate of 2500 rpm, corresponding to a linear speed of 13 m/s at the drum surface.

### 2.3. Analysis of Morphology and Crystallinity

The morphologies of the P(VDF-TrFE) nanofibers were examined by scanning electron microscopy (SEM, JSM-5800, JEOL, Akishima, Japan) at an accelerator voltage of 15 kV. Fast Fourier transforms (FFT) was performed on SEM images using the ImageJ software (National Institutes of Health, Bethesda, MD, USA) to determine the degrees of the nanofibers alignment. In order to analyze the compositions and crystallinities of the P(VDF-TrFE) nanofibers, Fourier transform infrared spectroscopy (FTIR, Nicolet iN10 MX, Thermo Fisher Scientific Inc., Waltham, MA, USA) in absorbance mode, and X-ray diffraction (XRD, D/MAX2200PC, Rigaku Corporation, Tokyo, Japan) over the 15–25° 2θ range were employed. A P(VDF-TrFE) spin-coated film was fabricated to study its diversity compared with the electrospun nanofibers. In addition, the P(VDF-TrFE) nanofibers were annealed at 130 and 140 °C for 2 h to investigate the variations in β-phase crystallinity.

### 2.4. Measurement of the Piezoelectricity

The local piezoelectric response of a single P(VDF-TrFE) nanofiber was evaluated using piezoresponse force microscope (PFM) (MFP-3D, Asylum Research, Santa Barbara, CA, USA). A conductive Ti/Ir coated Si cantilever (ASYELEC-01, Asylum Research, Santa Barbara, CA, USA) with a force constant of 2 N/m and a free air resonance frequency of 70 kHz was used during the measurements. P(VDF-TrFE) nanofibers were sparsely collected on an ITO-coated glass substrate using the electrospinning method. The vertical piezoresponse hysteresis loops were measured by dual ac resonance tracking PFM (DART-PFM) mode. The contact frequency varied from 275 to 285 kHz due to slightly changes on sample surface from point to point. An alternate voltage of 2 V was applied between the PFM cantilever tip and the bottom electrode. The nanodomain switching characteristics of individual nanofibers with nanometric resolution were studied by switching spectroscopy PFM (SS-PFM) measurement. For SS-PFM, a sequence of pulses DC bias voltage (Vdc) was utilized to offer the nanofiber with essential electric field. The amplitude of the DC pulses varied stepwise from −50 to 50 V. The PFM amplitude and phase measurements were carried out in the “off state” of the pulses, so that the electrostatic effect was eliminated from the hysteresis. In the process, at least three different locations along the individual fiber were examined.

## 3. Results and Discussion

### 3.1. Morphologies and Crystallinities of P(VDF-TrFE) Nanofibers

The far-field electrospinning method using a rotating-drum collector can produce well-aligned P(VDF-TrFE) nanofibers. The SEM images displayed in Figure 1b,c show that the nanofibers are distributed uniformly and aligned at the micron and millimeter levels. Figure 1d presents quantitative information on the degree of nanofiber alignment by applying FFT to SEM micrographs. In addition, the diameters of nanofibers studied here are mainly in the 400–550 nm range, with a distribution shown in Figure 1e.

**Figure 2:**
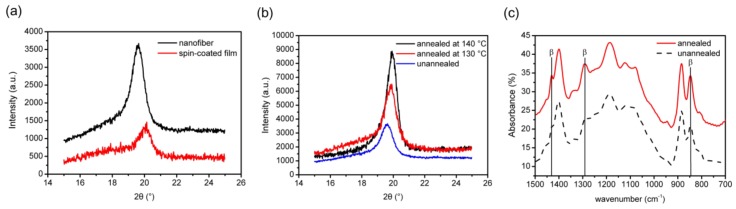
Characterization of the P(VDF-TrFE) nanofibers. (**a**) X-ray diffraction (XRD) patterns of the spin-coated P(VDF-TrFE) film and electrospun P(VDF-TrFE) nanofibers. (**b**) XRD patterns of the P(VDF-TrFE) nanofibers before and after annealing at 130 and 140 °C for 2 h. (**c**) Fourier transform infrared (FTIR) spectra of the P(VDF-TrFE) nanofibers before and after annealing at 140 °C for 2 h.

The piezoelectricity of P(VDF-TrFE) depends on the concentration of the piezoactive β-phase, which exhibits the best piezoelectric properties because of its large spontaneous polarization. Ferroelectric-polymer polymorphs are generally identified and quantified by XRD and FTIR. X-ray diffraction reveals the changes that occur in the crystalline structure of the P(VDF-TrFE) as a result of annealing, while FTIR is an important method for the evaluation of the polar β-phase content, α to β transitions, as well as the dipolar orientation in the material. XRD and FTIR spectra confirmed the formation of the β-phase crystalline structure in the P(VDF-TrFE) nanofibers as shown in Figure 2. In particular, the main peak at 2θ ≈ 19−20° in the XRD spectra corresponds to diffraction in (110) plane of the β-phase (Figure 2a), which means the as-spun P(VDF-TrFE) nanofibers contain a high content of the β-phase, whereas the spin-coated P(VDF-TrFE) film is required to be annealed to achieve a high β-phase fraction (Figure S1). Furthermore, annealing at a higher temperature improves the content of β-phase as shown in Figure 2b. The peaks associated with the β-phase (1431, 1285, and 846 cm^−1^) are highlighted in the FTIR spectra in Figure 2c.

### 3.2. PFM Characterization of the P(VDF-TrFE) Nanofiber

**Figure 3:**
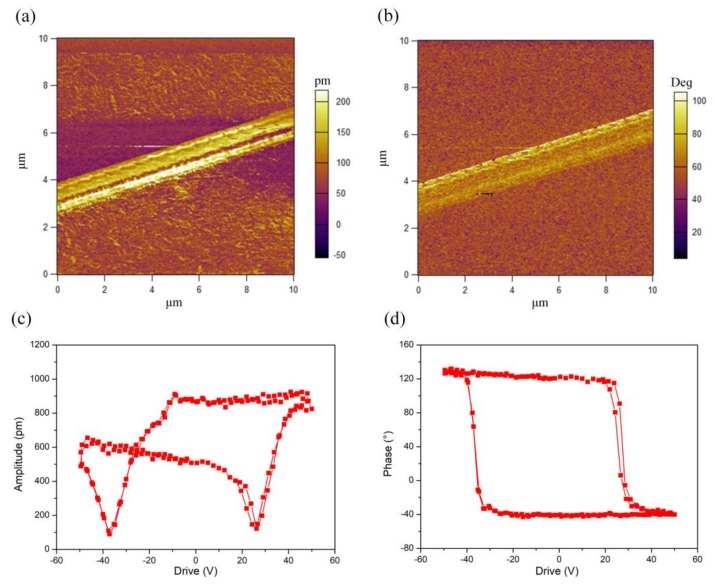
Piezoresponse force microscopy (PFM) images of a single P(VDF-TrFE) nanofiber. (**a**) Out-of-plane PFM amplitude image, revealing relatively uniform piezoelectric displacements over the entire nanofiber. (**b**) Out-of-plane PFM phase image, suggesting a uniform piezoelectric force response. (**c**) PFM amplitudes of the P(VDF-TrFE) nanofiber as functions of DC bias with two cycles, and (**d**) PFM Phases of the P(VDF-TrFE) nanofiber as functions of DC bias with two cycles, indicating a good repeatability for forward and reverse scans.

The piezoelectricity of the P(VDF-TrFE) nanofiber was characterized by the PFM method. As shown in Figure 3, the piezoelectric response of an individual P(VDF-TrFE) nanofiber annealed at 135 °C for 2 h was measured in contrast to the P(VDF-TrFE) nanofiber without annealing (Figure S2). In the experiment, a 10 μm × 10 μm area containing a P(VDF-TrFE) nanofiber was scanned. The local piezoelectric responses are displayed in Figure 3a,b. The amplitude image shows a strong piezoelectric contrast because of the deflection caused by the applied AC field. The phase image of the nanofiber clearly displays both the negative and positive values indicating antiparallel ferroelectric nanodomains with 180° domain walls. The local hysteresis loops under the effect of electric fields were evaluated by the PFM phase and amplitude loops, respectively (Figure 3c,d). The P(VDF-TrFE) nanofibers shows butterfly-shaped curves of amplitude versus Vdc loops. The forward and reverse coercive voltages are 26 and −37 V. The highest amplitude of piezoelectric signal reached 915 pm at the DC bias of 44 V shown in Figure 3d, the PFM phase of the P(VDF-TrFE) nanofiber undergoes a square-shaped hysteresis loops, which exhibits 180° switch of dipole moments about the chain axis between the applied positive and negative biases. It is noticeable that both the amplitude-voltage loops and the phase-voltage loops have good repeatability. Piezoresponses acquired from the P(VDF-TrFE) nanofiber show that the direction of electrical dipoles lies normal to the surface of the substrate [39]. Besides, the excellent piezoelectricity in this direction is due to the high β-phase content and well-alignments of electrical dipoles. 

### 3.3. Piezoelectric Out in the Longitudinal Direction 

**Figure 4:**
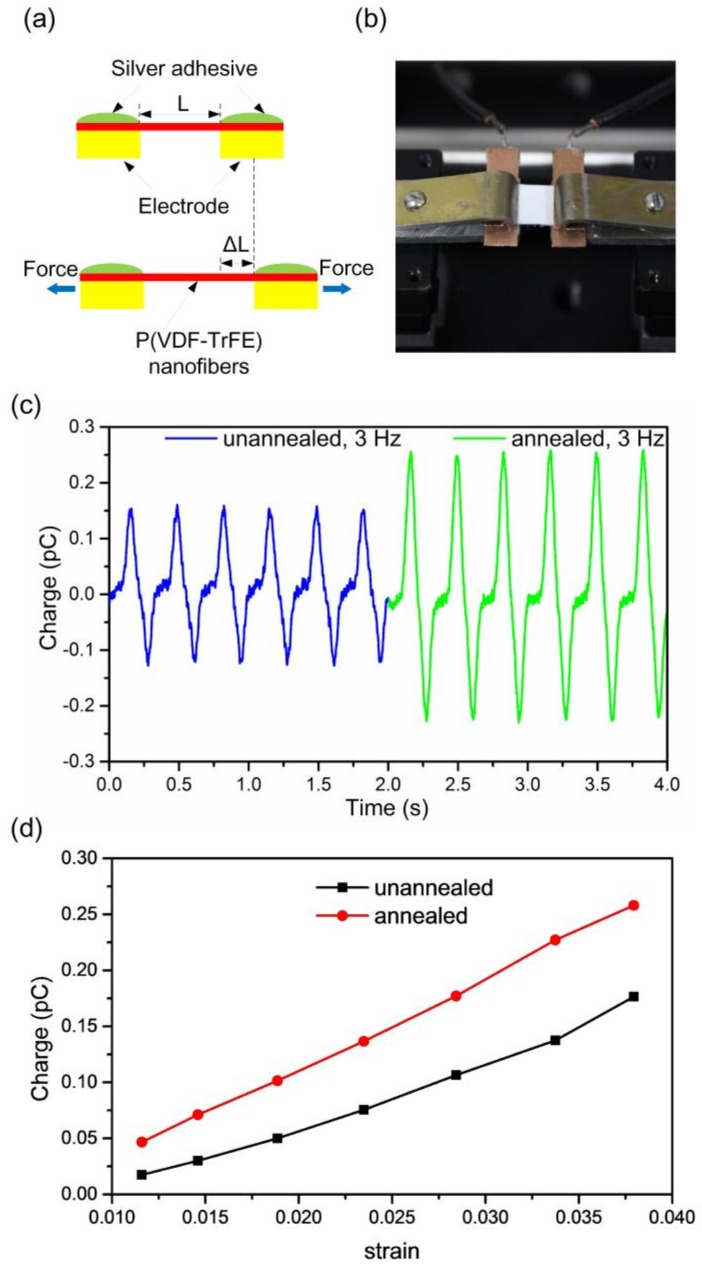
Piezoelectric output measurements in the nanofiber’s longitudinal direction. (**a**) Illustration of the electrical and stretching directions. (**b**) Illustration of the measurement setup. (**c**) Piezoelectric charge output under a tensile strain of 3.8% at 3 Hz for as-spun and annealed P(VDF-TrFE) nanofibers. (**d**) Piezoelectric charge outputs as functions of amplitude of strain for as-spun and annealed P(VDF-TrFE) nanofibers.

The piezoelectric output of the P(VDF-TrFE) nanofibers in the longitudinal direction was determined by applying tensional stress in the same direction. As shown in Figure 4a, electrospun P(VDF-TrFE) nanofibers were fixed across two copper electrodes with a gap of 4.5 mm. The nanofibers were mechanically fixed and electrically connected using silver adhesive. As illustrated in Figure 4b, one end of the P(VDF-TrFE) nanofiber mat was fixed, while the other end was connected to a movable clamp that was driven by a piezoelectric vibrator. The measurement setup is schematically illustrated in Figure S3. The driving signal from the function generator was amplified by a power amplifier. The piezoelectric charge output was measured by a charge amplifier. After noise reduction by a low-pass filter with a cut-off frequency of 20 Hz, the signals were recorded on a digital oscilloscope.

The piezoelectric output was measured for nanofibers with different heat treatments and stretching conditions. As shown in Figure 4c, the annealing process increases the charge output for a constant tensile strain. As illustrated in Figure 4d, the charge output increases by increasing the strain for both the as-spun and annealed samples. The as-spun P(VDF-TrFE) nanofibers deformed with a 3.8% amplitude strain generates an output charge of 0.16 pC. In this case, the Young’s modulus of P(VDF-TrFE) is assumed to be 340 MPa [30], the applied strain along the longitudinal axis of the nanofibers is 3.8%, and the effective contact area is determined from the cross-sectional area of the P(VDF-TrFE) nanofibers mat and its porosity, which are 0.57 mm^2^ and 65%, respectively. As a result, the piezoelectric constant in the nanofiber’s longitudinal direction is calculated to be 0.04 pC/N. The weak piezoelectric effect in the nanofiber’s longitudinal direction further confirms that the direction of polarization of the P(VDF-TrFE) nanofibers fabricated by the far-field electrospinning process is normal to the surface of the substrate.

### 3.4. Directional Strain Sensing by Aligned Nanofibers Mats

**Figure 5:**
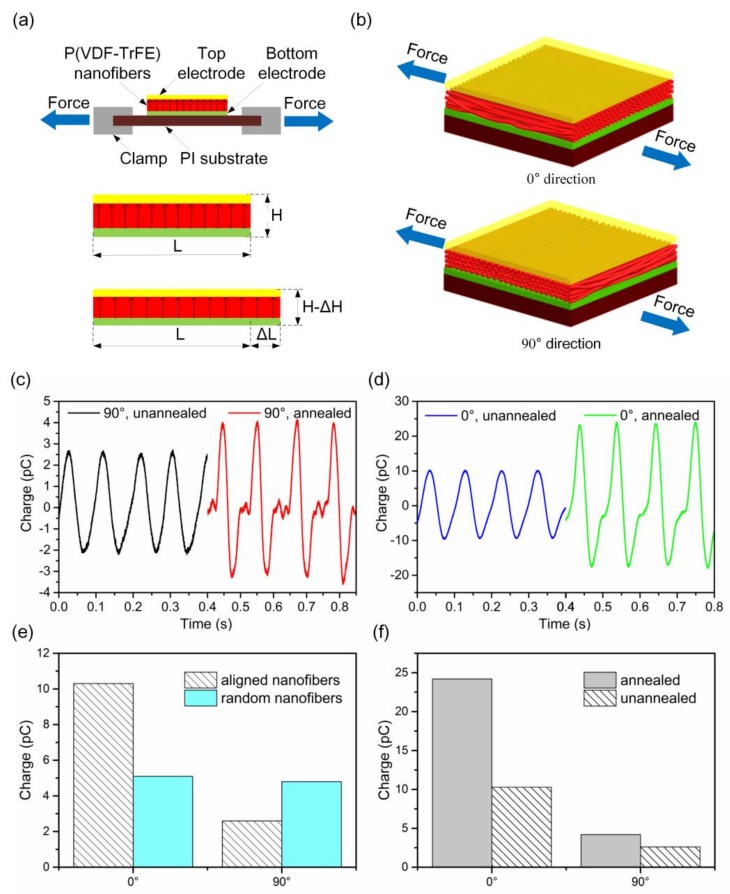
Directional strain sensing measurements using the P(VDF-TrFE) nanofiber mat. (**a**) Cross-sectional view and (**b**) **t**op view of the measurement setup. (**c**) Piezoelectric charge outputs with alternating strain in the 90° direction. (**d**) Piezoelectric charge outputs with alternating strain in the 0° direction. (**e**) Piezoelectric charge outputs for devices with aligned and random nanofibers that stretched in 0° and 90° directions, indicating directional sensing ability of well-aligned nanofibers. (**f**) Piezoelectric charge outputs for devices with annealed and unannealed nanofibers that stretched in 0° and 90° directions, indicating enhanced piezoelectric response due to annealing process.

A strain sensing device composed of P(VDF-TrFE) nanofibers sandwiched by electrodes was fabricated. The upper and lower electrodes are both 7 mm square. The thickness of the P(VDF-TrFE) nanofibers mat is approximately 50 µm. The sensor device was positioned with two clamps that were driven by a piezoelectric vibrator as shown in Figure 5a. When the device was stressed in the direction parallel to the surface of the P(VDF-TrFE) nanofibers mat, it became compressed by ΔH in the direction normal to the mat surface due to Poisson’s ratio. As shown in Figure 5b, the stretching direction aligned with the nanofibers themselves was defined as the “0° direction”, while the stretching direction perpendicular to nanofiber alignment direction in the plane of the nanofiber mat was defined as the “90° direction”.

As shown in Figure 5c,d, the charge output as a function of strain applied in the 0° direction reached 10 pC for the device fabricated from well aligned as-spun nanofibers. The P(VDF-TrFE) nanofibers mat showed a much higher charge output with strain in the 0° direction than in the 90° direction. It indicates the aligned P(VDF-TrFE) nanofibers mat exhibits enhanced directional strain sensing properties. However, randomly oriented P(VDF-TrFE) nanofibers fabricated by far-field electrospinning (FFES) showed comparable charge outputs in both the 0° and 90° directions as shown in Figure 5e. The strain sensing device fabricated from the well-aligned P(VDF-TrFE) nanofibers mat exhibited much higher strain sensitivity than the device with random oriented nanofibers. The directional strain sensing ability of the aligned nanofibers mat originated from its high elastic constant in the longitudinal nanofiber (0°) direction compared with the orthogonal (90°) direction. When the strain was applied in the 0° direction, almost all the nanofibers underwent deformation, leading to high charge outputs. In contrast, when the strain was applied in the 90° direction, the main effect was the increase or decrease of the distance (space) among nanofibers, and only a few nanofibers actually deformed. As shown in Figure 5f, higher piezoelectric charge outputs were obtained for the annealed samples in both 0° and 90° directions because annealing increases crystallization of the β-phase and the alignment of the electrical dipoles.

**Figure 6:**
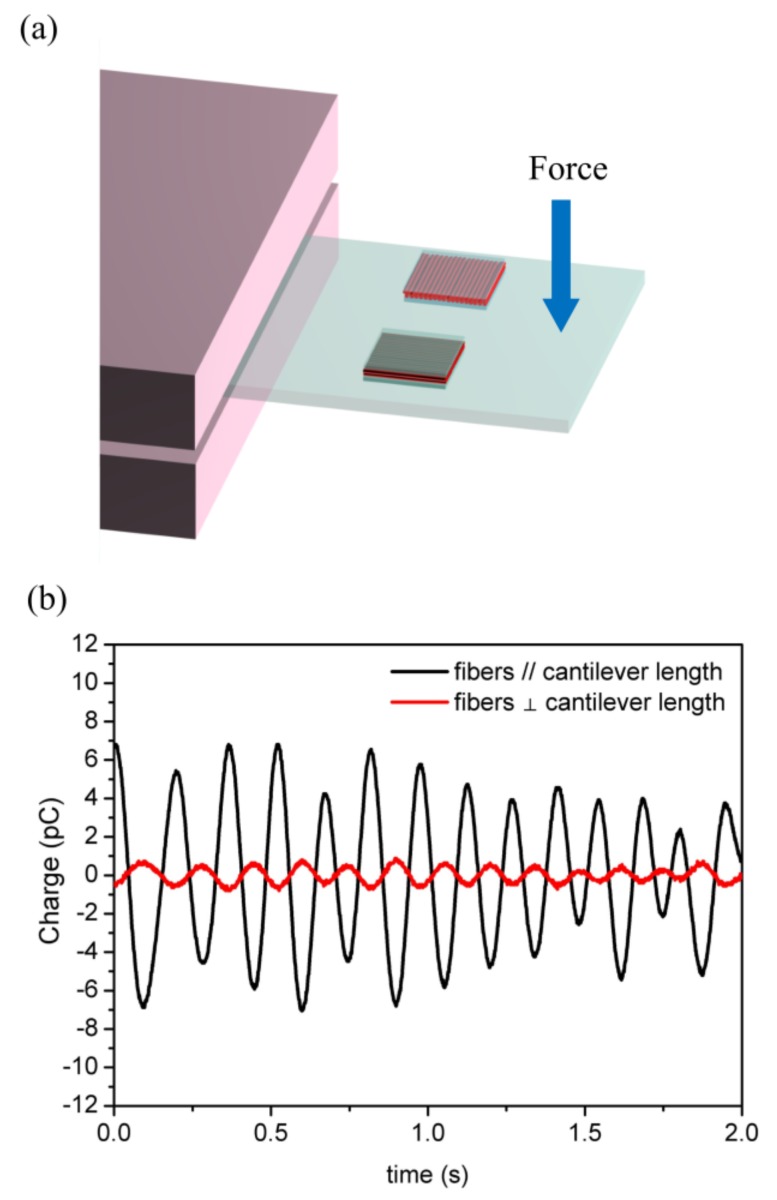
(**a**) Schematic illustration of the directional strain-sensing measurements. (**b**) Piezoelectric charge outputs with their fiber alignment directions parallel and perpendicular to the cantilever length direction.

In order to further demonstrate the directional sensing performance of the nanofibers, two P(VDF-TrFE) nanofiber mats with identical dimensions were embedded into a polydimethylsiloxane (PDMS) cantilever with their fiber alignment directions parallel and perpendicular to the cantilever length direction, respectively (Figure 6a). While the PDMS cantilevers vibrated, the nanofiber mat with their fiber alignment parallel to the cantilever’s length direction showed approximately five times higher piezoelectric charge output than the other one as shown in Figure 6b, suggesting an excellent directional strain sensing ability of the P(VDF-TrFE) nanofiber mats.

The PFM results presented here indicates that the electrospinning process for P(VDF-TrFE) nanofibers yields high population of aligned electrical dipoles in the direction normal to the substrate surface, thereby demonstrating excellent piezoelectric response without further electrical poling process. Compared with spin-coated P(VDF-TrFE) films, which require additional electrical poling to achieve high piezoelectricity [40], the P(VDF-TrFE) nanofibers are advantageous for excellent response without annealing. As shown in Figures 3c and 5f, the annealing process significantly enhanced the piezoelectric response, which was verified by previous studies for both PVDF and P(VDF-TrFE) [41,42,43]. Directional sensing is universal in biological sensory organs such as hair cells in the inner ear, lateral line neuromasts of fish, and mechanosensitive hair sensilla of insects [44,45,46]. Well-aligned P(VDF-TrFE) nanofibers are promising materials to mimic the directional strain sensing functions with high mechanosensitivity. 

## 4. Conclusions

In summary, far-field electrospinning combined with a rotating-drum collector was demonstrated to be an efficient method for the production of highly aligned P(VDF-TrFE) nanofibers. XRD and FTIR spectra confirmed that the as-spun P(VDF-TrFE) nanofibers (without mechanical stretching or electrical poling) were composed of high proportion of piezoactive β-phase and could be further enhanced by annealing. Specifically, the PFM experiments and the piezoelectric output in the nanofiber’s longitudinal direction demonstrated that the electrical dipoles preferred to orient themselves in the direction normal to the nanofiber mat surface. We developed well-aligned P(VDF-TrFE) nanofibers mats that exhibited enhanced directional strain sensing properties. These findings provide a new strategy for optimizing the design and fabrication of biomimetic sensors using P(VDF-TrFE) nanofibers.

## Supplementary Materials

The supplementary materials are available online at http://www.mdpi.com/2073-4360/10/4/364/s1.
