# Supplementary material for: Aligned P(VDF-TrFE) Nanofibers for Enhanced Piezoelectric Directional Strain Sensing
Source: Polymers (Basel). 2018 Mar 25;10(4):364. doi: 10.3390/polym10040364 (PMC6414850; doi:10.3390/polym10040364)
Supplement: Supplementary file 1 [file polymers-10-00364-s001.pdf]

Supplementary Information

Aligned P(VDF-TrFE) Nanofibers for Enhanced Piezoelectric Directional Strain Sensing

Yonggang Jiang [a], Longlong Gong [a], Xiaohe Hu [a], Yong Zhao [b], Huawei Chen [a],

Lin Feng [a, c, *], Deyuan Zhang [a]

[a] Institute of bionics and Micro-Nano sytems, Beihang University, Beijing, 100191, P.R. China.

[b] Laboratory of Bio-inspired Smart Interfacial Scienece and Technology of the Ministry of Education, School of Chemistry, Beihang University, Beijing, 100191, P.R. China.

[c] International research institute of Multidisciplinary Sciencem, Beihang University, Beijing, 100191, P.R. China

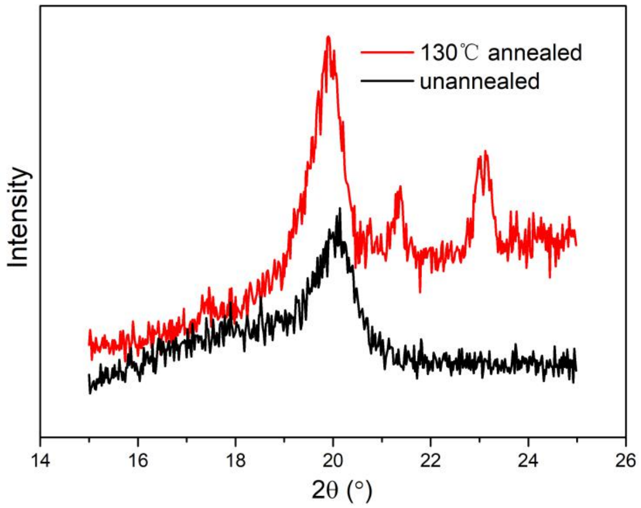

Fig. S1. XRD analysis results of spin-coating P(VDF-TrFE) films, indicate that annealing treatment contributes to the formation of β-phase in spin-coating P(VDF-TrFE) film.

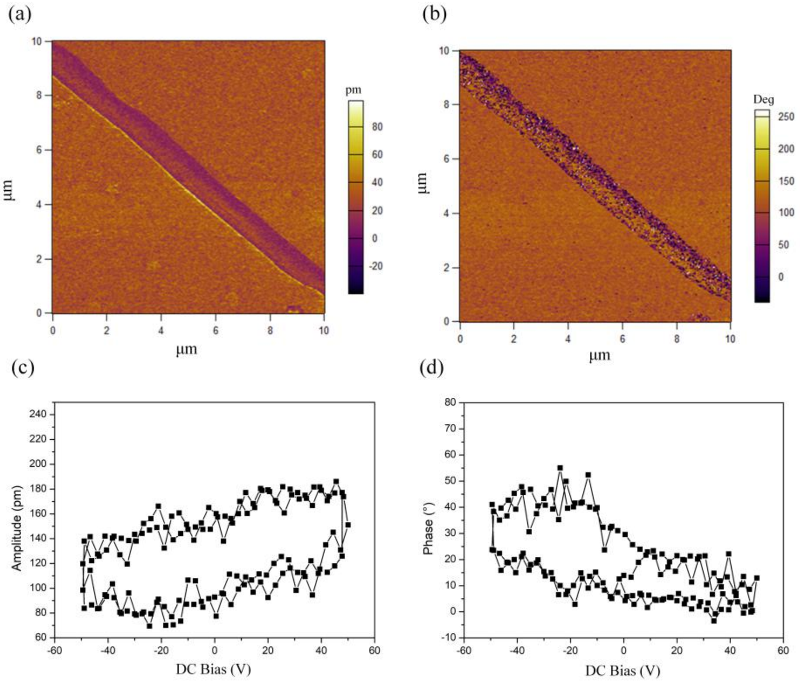

Fig. S2. PFM images of P(VDF-TrFE) nanofiber without annealing. (a) Out-of-plane PFM amplitude image and (b) PFM phase image of P(VDF-TrFE) nanofiber with 2 V driving voltage. (c) PFM amplitude and (d) PFM phase of the P(VDF-TrFE) film varying with the DC bias in single point measurements.

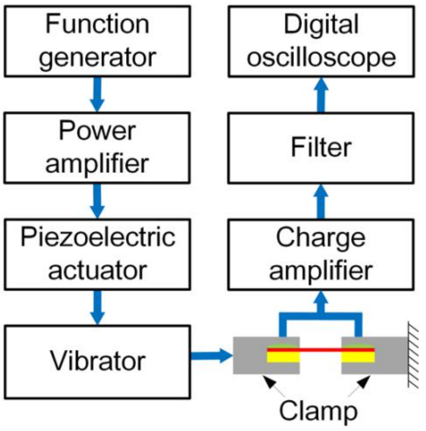

Fig. S3. Schematic illustration of the measurement setup for piezoelectric output in the nanofiber's longitudinal direction. The P(VDF-TrFE) nanofiber mat was fixed to a movable clamp that was driven by a piezoelectric vibrator. In order to drive the piezoelectric actuator, a power amplifier was used to amplify the signal from a function generator. The piezoelectric output charge was measured by a charge amplifier, and a low-pass filter was used for noise reduction.
